# S6K2: The Neglected S6 Kinase Family Member

**DOI:** 10.3389/fonc.2013.00191

**Published:** 2013-07-24

**Authors:** Olivier E. Pardo, Michael J. Seckl

**Affiliations:** ^1^Division of Cancer, Department of Surgery and Cancer, Imperial College, Hammersmith Hospital, London, UK

**Keywords:** S6K2, S6 kinase, selectivity, specificity, function, RPS6KB2, cancer

## Abstract

S6 kinase 2 (S6K2) is a member of the AGC kinases super-family. Its closest homolog, S6K1, has been extensively studied along the years. However, due to the belief in the community that the high degree of identity between these two isoforms would translate in essentially identical biological functions, S6K2 has been largely neglected. Nevertheless, recent research has clearly highlighted that these two proteins significantly differ in their roles *in vitro* as well as *in vivo*. These findings are significant to our understanding of S6 kinase signaling and the development of therapeutic strategies for several diseases including cancer. Here, we will focus on S6K2 and review the protein–protein interactions and specific substrates that determine the selective functions of this kinase.

## Introduction

The ribosomal protein S6 kinases constitute a super-family of proteins initially discovered based on their ability to phosphorylate a 40S ribosomal subunit component, the ribosomal S6 protein. The p90 ribosomal S6 kinases (RSKs), comprising RSK1–4 (1), were first identified followed by the p70 ribosomal S6 kinase, S6K1 (2, 3). It took an additional 10 years for the p70 ribosomal S6 kinase homolog, S6K2, to be discovered (4–6). The high degree of homology between S6K1 and S6K2 has for many years led researchers to assume that these were redundant kinases with essentially overlapping functions. This introduced a bias toward S6K1-oriented research, as this isoform came to be considered the prototypical S6K. However, more recent research clearly indicates that these two isoforms also have distinct biological functions the understanding of which may have implications for therapeutic intervention. Therefore, while other publications exist that review S6Ks and their upstream pathways (7, 8), here we will focus specifically on S6K2 and highlight the distinct biological function of this isoform.

## Structure of S6K2

**Figure 1:**
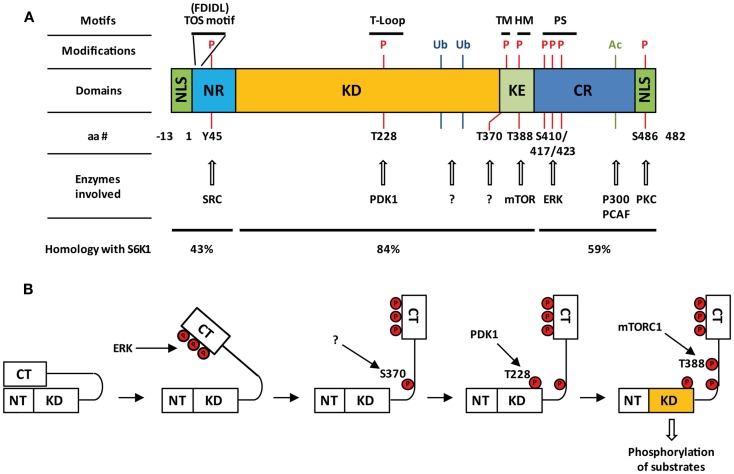
**Structure and activation of S6K2**. **(A)** Domain organization of S6K2, post-translational modifications together with involved enzymes, and percentage homology with S6K1. Nuclear localizations sequences (NLS); N-terminal regulatory region (NR); kinase domain (KD); kinase extension region (KE); C-terminal regulatory region (CR); pseudo-substrate domain (PS); turn motif (TM); hydrophobic motif (HM); pseudo-substrate region (PS); phosphorylation (P), ubiquitination (Ub); acetylation (Ac). **(B)** Step-wise model of activation of S6K2.

Human S6K2 is encoded by the 15 exons of the *RPS6KB2* gene on chromosome 11 (11q13). The S6K2 mRNA (ID ENST00000312629) gives rise to two protein products through the use of alternative translational start sites: a long form (p56 S6K2) and a short form (p54 S6K2) that differ by the presence or absence of an N-terminal 13 amino acid segment. The overall structure of S6K2 is very close to that of S6K1 (Figure 1A). The kinase domain of S6K2 shares 83% amino acid identity with that of S6K1, a fact that has long justified the lack of interest in finding isoform-specific substrates for these proteins. The kinase domain is followed toward the C-terminus by a kinase extension domain and a pseudo-substrate inhibitory region. The greatest degree of divergence between S6K1 and S6K2 lies in the C-and N-terminus, a fact that has enabled the development of S6K2-specific antibodies (9). The presence in the C-terminus of S6K2 of a nuclear localization sequence (NLS) means that this isoform is predominantly localized to the nuclei of quiescent cells (10). In addition, the long form of S6K2 contains in its 13 amino acid extension an additional putative NLS. This results in the different cellular distribution of these two isoforms as the two NLS motifs in p56 S6K2 confers constitutive nuclear localization to this variant, while p54 S6K2 shuttles between the nucleus and the cytoplasm in response to growth factor signaling. The C-terminus of S6K2 also contains a proline-rich region which has been proposed to promote interaction with SH3 and WW domains putatively present in its binding partners (4). While shorter isoforms of S6K1 have been shown to be generated by alternate mRNA splicing (11), no such variants have yet been reported for S6K2. However, the high degree of conservation between the two proteins raises the possibility that similar regulation may take place for the *RPS6KB2* gene. Indeed, eight transcripts have been reported for S6K2 with a corresponding protein found for only one (ID ENST00000312629) of the four protein coding transcripts (ID ENST00000539188, ENST00000524934, ENST00000524814, ENST00000312629). This may have important functional consequences as, unlike its full length counterpart, an S6K1 splice variant, p31S6K1, was shown to have oncogenic potential (12).

## S6K2 Activation and Post-Translational Modification

### S6K2 activation

Many of the residues that are required for kinase activation are common between S6K1 and S6K2 as seven of the eight serine/threonine phosphorylation sites present on S6K1 are conserved in S6K2 (Thr-228, Ser-370, Thr-388, Ser-403, Ser-410, Ser-417, and Ser-423 on p54 S6K2) (4, 6, 10) (Figure 1A). The activation of S6K2 occurs in a step-wise manner (Figure 1B). An initial barrier to overcome is the repression exerted by the C-terminal autoinhibitory pseudo-substrate domain. This is dealt with by phosphorylation of the three proline-directed serines in the autoinhibitory domain, Ser-410, Ser-417, and Ser-423 downstream of MEK/ERK signaling. We and others have found this first step to be crucial for S6K2 activation in various cell types (13, 14), as this domain exerts a far more repressive role on S6K2 activity than it’s equivalent for S6K1 (15, 16). This event is presumed to open the kinase conformation, exposing additional phosphorylation sites to activating kinases. In agreement with this, deletion of the autoinhibitory region increases basal activity of S6K2 and sensitizes the kinase to activation by various agonists (15). Subsequent phosphorylation of Ser-370 then enables phosphorylation of Thr-388 by the mTORC1 complex followed by that of Thr-228 by PDK1 (17). The T388 site lies within a conserved sequence of the kinase extension domain (F-X-X-F/Y-S/T-F/Y) known as the hydrophobic motif, a region found in many AGC kinases. Phosphorylation of this site by mTOR is achieved following the binding of the mTORC1 complex component Raptor to the TOR signaling (TOS) motif present in both S6K1 and 2 (18, 19). Interestingly, despite the conservation of the hydrophobic motif, substitution of Thr-388 by a glutamic acid (T388E) renders S6K2, but not S6K1, constitutively active. However, phosphorylation of both the Ser-370 and Thr-228 is crucial for S6K2 activity. Indeed, substitution of the latter site for alanine renders the T388E mutant inactive while that of the first prevents Thr-388 phosphorylation. As S6K2 is mainly a nuclear protein and mTOR shuttles between the cytoplasm and the nucleus, it was shown that S6K2 activity was increased by targeting mTOR expression to the nucleus (20).

**Figure 2:**
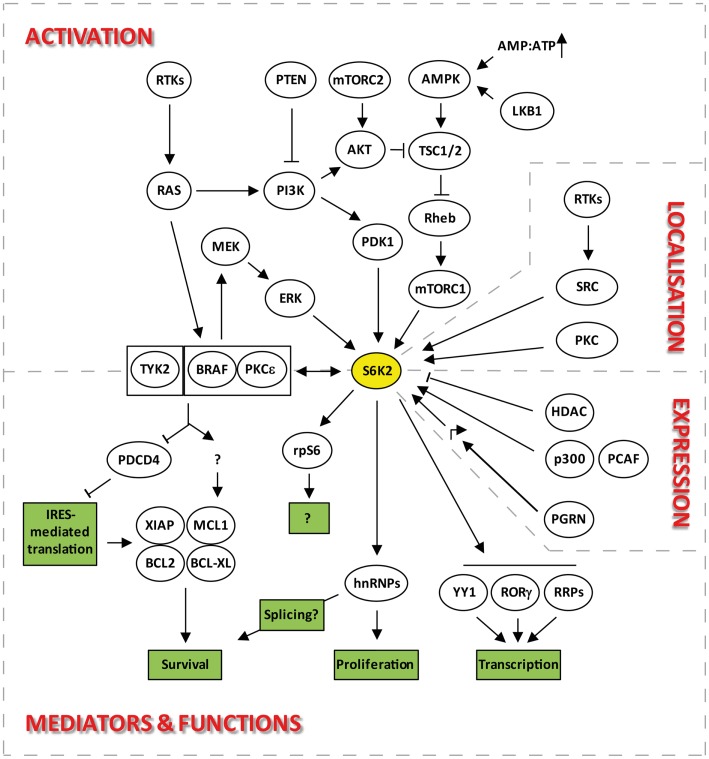
**Signaling pathways upstream and downstream of S6K2 that regulate its activation, localization, expression, and functions**.

Despite S6K1 and S6K2 both lying downstream of mTOR (Figure 2), there is evidence to indicate that they may be regulated through different pools of this upstream kinase. Indeed, both S6K isoforms react differently to nutrient deprivation, a known modulator of mTOR activity. For instance inhibition of protein synthesis by leucine deprivation in myotubes, results in dephosphorylation of S6K1, without affecting S6K2 activity (21). The existence of two separate pools of mTOR regulating the two S6K isoforms is further suggested by the differential sensitivity of S6K1 and 2 kinase activity to the mTOR inhibitor, rapamycin. Indeed, the involvement of mTORC1 in the activation of S6K2, led several researchers to report the sensitivity of S6K2 to this inhibitor (14, 17, 22). However, the majority of reports suggesting equivalent sensitivity of S6K1 and 2 to rapamycin used concentrations of this drug that non-selectively inhibit the MEK/ERK pathway, therefore indirectly targeting S6K2 independently of its effect on mTOR (13). Hence, when used at the minimal concentrations that fully inhibit S6K1 activity, rapamycin often fails to significantly alter S6K2 activity in several cell systems [(13) and unpublished data from our lab]. These findings are consistent with the reported existence of a rapamycin-resistant mTORC1 activity pool (23, 24) that can efficiently be targeted by mTOR ATP-competitive inhibitors (23, 25).

### Additional phosphorylation events

While expression of an mTOR variant targeted to the nucleus increases activation of S6K2, nuclear localization of S6K2 is not indispensable for activation of this kinase. Indeed, S6K2, but not S6K1, is phosphorylated *in vitro* as well as *in vivo* by protein kinase C (PKC) (26). The site of phosphorylation was identified as S486 in p54 S6K2 (S486 in p56 S6K2), located within the C-terminal NLS. While phosphorylation of this site did not affect the activity of S6K2, it impaired the function of the NLS leading to cytoplasmic accumulation of the kinase upon cell stimulation with PKC agonists such as PMA. In contrast, S6K1 sub-cellular localization was not modulated by this treatment, highlighting a specific mechanism regulating S6K2 nucleo-cytoplasmic shuttling. All PKC isoforms were capable of phosphorylating S6K2, with PKCδ appearing to be the most efficient *in vitro*. However, this specificity seemed to disappear *in vivo* with all PKCs being equally potent.

In addition to being serine/threonine phosphorylated, S6K2, as well as S6K1, can be tyrosine phosphorylated downstream of receptor tyrosine kinase activation (27). Both S6Ks were found to associate with the PDGFR, HGFR, and CSFR. Upon stimulation of these receptors, N-terminal tyrosine phosphorylation of S6Ks occurred (Y39 on S6K1 and Y45 on S6K2) in a SRC-dependent manner. This event did not result in modulation of S6Ks activity or the gross redistribution of these enzymes, although a fraction of S6K1 was found to relocates to membrane ruffles where the activated RTKs are expected to reside. Although this pathway seems shared between S6K1 and 2, it is worth noting that while SRC family members were equally able to phosphorylate both isoforms *in vitro*, S6K2, but not S6K1, was tyrosine phosphorylated in response to FYN transgene expression *in vivo*. This may reflect differential wiring of these isoforms to the SRC family members through alternate cellular multi-protein complexes.

In addition to phosphorylation events, S6K2 is also the target of ubiquitination (28, 29) and of acetylation on a lysine residue close to the C-terminal PDZ binding motif (30). The latter modification does not impact on S6K2 kinase activity or sub-cellular localization but increases the stability of this kinase (see Control of S6K2 Steady-State Levels).

## Cellular Expression and Localization of S6K2

### Control of S6K2 steady-state levels

There is currently little to no information on the transcriptional or translational regulation of S6K2 expression. However, much more is known about the regulation of S6K2 stability. Steady-state levels of S6K2 and 1 are regulated through the opposing effects of ubiquitination and acetylation. Indeed, degradation of S6K1 and S6K2 is mediated by ubiquitination followed by proteosomal degradation (28, 29). This is promoted by growth factor signaling in cell lines although independently of phosphorylation/activation of these kinases. Conversely, cell stress, such as that induced by UV exposure, stabilizes both proteins. However, unlike for S6K1, the molecular pathway regulating S6K2 ubiquitination is currently unknown. Indeed, while the ROC1 ubiquitin ligase was shown to specifically interact and ubiquitinate S6K1 (31), the corresponding partner for S6K2 has not yet been identified. S6K1/2 degradation is counteracted by the stabilization of this protein through a C-terminal lysine acetylation event. This occurs through interaction with the acetyltransferases p300 and p300/CBP-associated factor (PCAF) (Figure 2). Hence, overexpression of p300 or inhibition of deacetylases leads to an increase in the levels of both kinases (30). Interestingly, overexpression of p300 has been linked to decreased overall survival in patients suffering from a wide variety of malignancies (32–36) while that of PCAF has been associated with drug resistance (37–39). However, the role of S6K2 in these backgrounds still remains to be established. S6K1 seems to be targeted by both HDAC and sirtuins for de-acetylation, but S6K2 seems entirely dependent on HDAC showing differential regulation of these isoforms. The involvement of HDACs in this process may provide a further link between these kinases and the transcriptional machinery (see Regulation of Transcription). Moreover, the stabilization of S6K2 by acetylation together with the pro-survival and drug resistance phenotypes associated with overexpression of this kinase (see Control of Cell Survival) may hinder the efficiency of HDAC inhibitors in the clinic.

### Sub-cellular localization of S6K2

As mentioned above, S6K2 mainly resides in the nucleus of resting cells. Closer examination reveals that it is the long form of S6K2 that is predominantly nuclear by virtue of its two NLSs. In contrast, the short form of S6K2 shuttles between the nucleus and the cytoplasm in response to growth factor signaling. In addition to its diffuse nuclear localization, a proportion of S6K2, but not S6K1, has been shown to co-localize with CTR453 and γ-tubulin at the level of the centrosome. This localization, demonstrated in multiple cell lines using both immunofluorescence and immunoblotting of purified centrosome (40), was stable throughout the cell cycle. Finally, cytoplasmic S6K2 has been shown to have a speckled distribution (40), although the structural components of these speckles remains undetermined.

## Tissue Expression of S6K2 in Health and Disease

### Physiological expression of S6K2

S6K2 is expressed at various levels in different mouse and human tissues, and its expression levels often inversely correlate with those of S6K1 (41). In Humans, S6K2 expression was found in all tissues with the exception of the neuropil, the peripheral nervous system, and adipocytes. However, expression levels between organs vary considerably, with highest levels found in the gastrointestinal tract, the central nervous system and the lung. In contrast, most mesenchymal cells stain weakly for S6K2. Although S6K2 is detected in normal tissues, its expression levels are often very low compared to those found in corresponding tumor samples [(42) and see S6K2 Protein Levels in Cancer and Normal Corresponding Tissues].

### Expression of S6K2 in cancer and its significance

#### S6K2 protein levels in cancer and normal corresponding tissues

S6K2 has been shown to be expressed in the overwhelming majority (88%) of cancer samples investigated and the level for this kinase compared between several cancer types and corresponding normal tissue. These studies demonstrated that normal tissue usually express low levels of this kinase as compared to those found in tumor samples (42–45) and that overexpression of S6K2 was more common than that of S6K1 (e.g., 80 versus 25% in breast and 18 versus 8% for endometrial cancer). In addition to changes in the levels of expression of S6K2, investigators also identified changes in the sub-cellular localization of this kinase between normal and malignant tissues. For instance, Filonenko et al. demonstrated that nuclear accumulation of S6K2 was a distinguishing feature of breast cancer cell *in situ*, whereas this kinase was only found in the cytoplasm of normal breast cells (43). Moreover, presence of S6K2 in the nucleus positively correlated with proliferating cell nuclear antigen (PCNA) and Ki-67 staining in breast cancer tissues, demonstrating a link between the presence of S6K2 in this compartment and cell proliferation (46). It is noteworthy that no such correlation existed in the case of S6K1. Interestingly, nuclear localization of S6K2 was further increased in cells localized at the periphery of the tumor where tumor cells are in contact with healthy tissue. Hence, it is tempting to speculate that the nuclear activity of S6K2 is somehow promoting tumorigenesis. The role of S6K2 in tumorigenesis is further suggested by the fact that expression levels of S6K2 in various tissues seem to influence the role of S6K1 in mediating PTEN haplo-insufficiency-driven tumorigenicity. Indeed, S6K1 downregulation impaired tumor development downstream of mTORC1 hyperactivation in *Pten*^±^ mice only in tissues where S6K2 expression levels were low (41). Furthermore, in endometrial cancer, increased nuclear localization of S6K2 correlated with tumor grade (44), while in lung cancer, increased expression of S6K2 correlated with drug resistance (42). This would further suggest that S6K2 expression is linked to cancer progression.

Interestingly, phosphorylation of the ribosomal S6 protein was shown not to correlate with expression levels of either S6K1 or S6K2 in endometrial and breast cancer (43, 44). This lack of correlation is in contradiction with the results obtained from animal models showing that S6K2 knockout mice, unlike their S6K1 counterpart, showed a dramatic reduction in the cellular levels of S6 phosphorylation (47). Hence, the lack of correlation found in tissue samples may be an artifact generated by the modalities of tissue processing or the saturation of these phosphorylation events beyond a certain threshold of S6K expression. However, conclusions on this issue are further complicated by results obtained in knock-in mice where the five phosphorylation sites in S6 are replaced by alanine residues. Indeed, these animals show a phenotypic overlap with that of S6K1^−/−^ mice including a cell growth defect associated with reduction in cell size (48). This is somehow surprising considering the lack of impact on S6 phosphorylation of knocking out S6K1 and the previously mentioned privileged link between S6K2 and S6 phosphorylation in mice (47), a finding that our lab corroborated in human cell lines using an siRNA approach (42). However, some reports suggest that reliance of S6 phosphorylation on one or the other S6K isoform may depend on the nature of the mitogen stimulation (49). Also, these discrepancies may be explained by date failure to take into account the sub-cellular localizations of S6K1, S6K2, and S6, variables that may impact considerably on the overall phosphorylation of the latter protein. The change in sub-cellular distribution of S6K2 observed between tumor and healthy tissues would support this hypothesis (43, 44). Finally, it may be postulated that increased expression of S6K1 and S6K2 does not correlate with increased activity of these kinases.

#### Amplification of S6K2 in cancer

Amplification of the chromosomal region 11q13 where the *S6K2* resides is found in 15–20% of breast cancers samples studied, an event implicated in resistance to endocrine therapy (50). Amplification of *S6K2* among these samples correlated with increased mRNA levels for this kinase, ER positive status and worse prognosis (45). However, *S6K2* copy number gain and nuclear localization of the protein was related to an improved benefit from tamoxifen among patients with ER+/PgR+ tumors, while in the ER+/PgR− subgroup, nuclear S6K2 rather indicated decreased tamoxifen responsiveness. The presence of the *S6K2* amplicon correlated significantly with amplification of the 8p12 region, containing the *FGFR1*, *PPAPDC1B*, and *4EBP1* genes (50). This latter amplification was associated with increased mRNA levels for all three genes. Of the genes present in the proximal 11q13 region, *S6K2* expression alone correlated with that of *FGFR1*, *PPAPDC1B*, and *4EBP1*. Using univariate analysis, it was found that 8p12 gain/amplification was significantly associated with increased risk of distant recurrence among patients with 11q13 positive tumors. This analysis could be further refined to demonstrate that high level of *FGFR1* and *4EBP1* mRNA expression alone predicted a worse outcome in this patient group. Although co-expression of FGFR1 and S6K2 would be logical in view of the link between FGF2-mediated survival and downstream S6K2 activity (42), an associated increased expression of 4EBP1, a reported tumor suppressor, may seem surprising. However, phosphorylated 4EBP1 has been suggested to stimulate mTORC1 activity (51), a process that would in turn increase S6K2 activation. In addition to the case of breast cancer, *S6K2*, but not *S6K1*, was found amplified in about 5% of gastric carcinoma patient samples (52). This amplification was associated with a significance decrease in the overall survival of patients with advanced disease.

#### S6K2 variants and disease

One of the neuropathological hallmarks in Alzheimer’s disease (AD) is the neurofibrillary tangles formed by hyperphosphorylated microtubule-associated Tau protein. Vazquez-Higuera et al. investigated genetic variations in a set of 20 candidates kinases involved in tau phosphorylation at sites correlating with AD (53). They reported that the distribution of the minor allele frequencies for these kinases did not differ significantly between sufferers and control groups, except for *S6K2* where variations in intron 2 was increased in patients (50%) versus controls (39%). In addition to correlating with increased risks of developing the disease, this minor allele was also associated with late onset of AD. Genetic variations in the *S6K2* gene have also been linked to the risk of developing colon cancer where they are found in tumors with SNPs in *PIK3CA*, CIMP positivity, and mutated KRAS2 (54). While CIMP positivity in colon cancer generally correlates with poor tumor differentiation and patient prognosis (55, 56), it was also shown to independently predict the survival benefit from 5-FU chemotherapy (57, 58). Hence, the polymorphism in *S6K2*, and its pathway associations, may have prognostic value and therapeutic significance. However, the true impact of these SNPs to S6K2 biological activity is yet to be experimentally confirmed.

## Biological Functions of S6K2

### Mouse models of S6K2 reveal little of its biological functions

Knockout mice for S6K1 and S6K2, in isolation and combination, have been bred (47). Single-knockout animals were viable and born at the expected Mendelian ratio. S6K1^−/−^ mice were significantly smaller than their wild-type counterpart while S6K2^−/−^ animals tended to be slightly larger. The latter change is thought to result from compensatory mechanisms whereby S6K2 knockout leads to increased S6K1 activity. This possibility is independently supported by cell line-based experiments in which RNAi-mediated silencing or inhibition of S6K2 leads to increased baseline S6K1 activity (13, 42). Taken together, these data provide confirmation for the proposed role of S6K1 in the control of cell size in mammalian cell lines (59). Conversely, S6K1 knockout mice presented with increased S6K2 mRNA levels in all organs tested, a fact that may explain the observed physiological inverse correlation between the tissue expression for S6K1 and S6K2 (see Physiological Expression of S6K2). In contrast to single-knockout animals, mice lacking both S6K1 and S6K2 suffered from perinatal lethality. This was not due to defects in cell cycle progression or 5′-TOP mRNAs translation in these animals. Also, analysis of embryos at 18.5 days of gestation indicated normal Mendelian distribution. Instead, one third of the double-knockout animals were born dead and the majority of those born alive developed signs of cyanosis leading to death shortly after birth. The minority of mice surviving past the first few days following delivery succeeded in reaching adulthood with similar growth rates as the S6K1^−/−^ animals and were fertile. Histopathological analysis performed to understand the reason for perinatal lethality amongst the double-knockout litters revealed no gross anatomical abnormalities. However non-viable animals showed hyperemic internal organs, occasional dilated heart chambers as well as several hemorrhagic sites. In short, none of the phenotypes observed in these animal models could be attributed to some distinct role of S6K2 and further biochemical analysis was required before biological functions of this kinase started to transpire.

### Regulation of transcription

Genome-wide microarray experiments revealed that S6K1 and 2 regulate the general transcriptional program. Indeed, 456 mRNAs were downregulated in the whole-cell extracts from starved S6K1/S6K2 double-knockout mice livers as compared to that of wild-type controls following re-feeding (60). Specifically, S6K2, but not S6K1, has been shown to directly interact in a mitogen-inducible fashion with the general transcription factor Yin Yang 1(YY1), an association that required the C-terminal region of S6K2 (61). YY1 has been involved in a wide range of biological processes through the recruitment of general components of the transcriptional machinery such as RNA polymerase II, ATF and SP1 as well as various transcriptional co-activators and co-repressors including DNA methyltransferases (DNMTs), histone acetyl transferases (p300, CBP, PCAF), histone deacetylases (HDAC 4), protein arginine methyltransferases (PRMTs), histone-lysine *N*-methyltransferase (Ezh1/2), Sumo-conjugating enzyme (Ubc9), and ubiquitin ligases (Mdm2). The function of YY1 is regulated by post-translational modifications including phosphorylation, sumoylation, acetylation, and ubiquitination so it is possible that interaction with S6K2 results in the phosphorylation and regulation of transcriptional activity of this protein. S6K2, along with S6K1, has also been involved in the transcription of ribosomal proteins (60). Indeed, analysis of the ribosome biogenesis transcriptional program after feeding in liver cells from S6K1^−/−^/S6K2^−/−^ mice showed that over 75 factors involved in ribosome biogenesis were controlled by S6Ks. Importantly, these changes were also observed in knock-in mice for a non-phosphorylatable mutant of rpS6. However, this was not associated with changes in the recruitment of RNAs into polysomes, revealing a role for S6Ks in the regulation of transcription that is dissociated from the translational program. Interestingly, S6K1 and 2 were functionally redundant in this biological process as overexpression of either isoform into the double-knockout cells rescued the phenotype.

The regulation of transcription by S6K2 also plays an important role in immune cells differentiation. Indeed, the import into the nucleus of RORγ, a critical transcription factor for the differentiation of a sub-class of IL-17-secreting CD4^+^ T-helper lymphocytes, is dependent on the binding of this protein to S6K2 (Figure 2) (62). This occurs because RORγ, a protein that lacks an NLS, uses S6K2 to piggyback its ways into the nucleus. The interaction of S6K2 with RORγ was resistant to rapamycin treatment. However, the import of RORγ into the nucleus was rapamycin-sensitive, suggesting a role for mTORC1 in S6K2-mediated nuclear import of RORγ.

### Regulation of protein translation

S6K2, like S6K1, appear not to be involved in the general protein translation program. Indeed, despite the impairment in S6 phosphorylation found in cells from S6K1/2 double-knockout mice, translation of 5′-TOP mRNAs were still promoted by mitogen stimulation (47). This and other data (63, 64) indicated that S6 phosphorylation is dispensable for this process., Furthermore, these results demonstrated that other kinases lying downstream of the MAPK pathway substituted for S6K1 and S6K2 in phosphorylating two serine sites (235 and 236) on S6 in response to mitogen stimuli. This role has since been attributed to members of the RSK family. However, unlike RSKs, S6Ks are capable of catalyzing the ordered phosphorylation of the five sites present in the C-terminus of the S6 protein (S236, S235, S240, S244, S247) (65), although the physiological significance of these events still remains unclear (66). While it is clear that S6K2 does not play a major role in the cap-dependent translation of housekeeping proteins, this kinase is involved in the cap-independent translation of selective mRNAs (Figure 2). These, including the Bcl-XL, MCL1, and XIAP mRNAs, may be selected for regulation by S6K2 through the presence in their 5′UTR of an internal ribosome entry site (IRES) (67, 68), the activity of which S6K2 controls through the phosphorylation of IRES transactivating factors (ITAFs) such as PDCD4 (69) (see Control of Cell Survival).

### Cell cycle regulation

S6K2 has been suggested to play a role in mitosis, as a pool of this kinase localizes at the centrosome and S6K2 activity peaks at the G2 and M phases of the cell cycle (70). However, it is worth noting that neither MEFs from S6K1/S6K2 double-knockout mice nor their corresponding embryonic stem cells show significant defects in cell proliferation or cell cycle distribution (47). Hence, the role of S6K2 in the cell cycle may be context dependent and limited to situations where mitogenic stimulus is applied or compensatory mechanisms are not available. Indeed, in IL3-dependent immortalized murine bone marrow-derived pro-B-cells and primary mast cells, IL3 stimulation activated S6K2, which contributed to the mitogenic effect of this growth factor (71). In these cells, S6K2 activity was able to shorten the G1 phase of the cell cycle, enabling cells to enter the S-phase at an increased rate. However, S6K2 activity alone was not able to substitute the need for IL3, as cells expressing a kinase-active S6K2 in the absence of IL3 failed to proliferate. This suggests that S6K2 is only playing a facilitating role in this process, a finding that may help explain the non-essential role of this kinase for cell cycle progression in animal models. It is worth noting that our recent collaborative research has highlighted specific S6K2 partners that mediate the effect of this kinase on the cell cycle. We showed that S6K2 binds several heterogenous ribonuclear proteins (hnRNPs) in a mitogen-inducible manner. One such RNP, hnRNPF was required for cell proliferation driven by S6K2 (72). Indeed, silencing of hnRNPF thwarted the proliferative effects of S6K2 while overexpression of this RNP increased cell proliferation in a rapamycin-sensitive manner. Consistent with the latter result, mitogen stimulation led to the recruitment of preformed S6K2-hnRNPF complexes to mTORC1.

The contradictory findings published on the role of S6K2 in the regulation of the cell cycle may also be explained by the extensive rewiring of signaling pathways occurring during tumorigenesis together with accompanying overexpression of S6K2. These may significantly change the contribution of this kinase to the promotion of mitosis, a possibility supported by the correlation between Ki-67 staining, a marker for cell proliferation, and S6K2 overexpression found in tumor samples (44). In addition, the increased nuclear localization of S6K2 in cancer (43, 44) may also impact on its pro-mitotic function. A possible increased involvement of S6K2 in the regulation of cell cycle in malignant tumors as compared to normal tissue could provide a therapeutic window for the targeting of this kinase in the treatment of cancer patients.

### Control of cell survival

The role of S6K2 in the regulation of apoptotic cell death was first demonstrated by research from our lab (42). The serum levels for basic fibroblast growth factor (FGF2) are often elevated in patients with a variety of malignancies and are a poor prognostic factor on uni- and multi-variate analysis (73– 78). We found that treatment of lung cancer cells with FGF2, used at concentrations commonly found in the serum of carcinoma patients, promoted cell survival, and drug resistance through translational up-regulation of anti-apoptotic proteins such as Bcl-XL and XIAP (79, 80). The mRNAs for these proteins are characterized by the presence in their 5′UTR of an IRES, a three-dimensional structure that represses the efficiency of their translation. Hence, increased expression of these proteins requires the unwinding of these structures and the recruitment of ITAFs that de-represses their translation. Investigation into the signaling involved in this response revealed that S6K2, but not S6K1, was required for the increased translation of these anti-apoptotic proteins in the absence of *de novo* mRNA synthesis (42, 80). In support of this, silencing of S6K2 using siRNAs prevented FGF2-induced drug resistance as well as up-regulation of Bcl-XL and XIAP. Moreover, in un-stimulated cells, S6K2 downregulation was accompanied by a decrease in the steady-state levels of both anti-apoptotic proteins, suggesting that this kinase is not only involved in promoting their production downstream of pro-survival signaling but also participates to their baseline translation. In contrast, silencing S6K1 had no impact on either protein whether in the presence or absence of mitogen stimulation. Conversely, overexpression of a kinase-active mutant for S6K2, but not S6K1, increased the translation of Bcl-XL and XIAP, promoted baseline cell survival and induced drug resistance in the absence of FGF2 stimulation (42). The anti-apoptotic function of S6K2 was dependent on the FGF2-inducible formation of a multi-protein complex comprising S6K2, BRAF, and PKCε (Figure 2). Indeed, disruption of this complex through the silencing of BRAF or PKCε prevented the pro-survival activity of S6K2. The composition of this multi-protein interaction was selective as it did not include other PKC or RAF isoforms. Similarly, S6K1 was unable to form a complex with these S6K2 partners. Tandem affinity purification using S6K2 as bait in the presence and absence of FGF2 stimulation in HEK93 cells enabled the identification of downstream mediators that regulate the translation of S6K2’s anti-apoptotic targets. One such interactor, the tumor suppressor programed cell death 4 (PDCD4) that normally binds to the IRES of Bcl-XL and XIAP to repress their translation, is phosphorylated by S6K2 (69). This post-translational modification leads to the degradation of PDCD4 and subsequent derepression of Bcl-XL and XIAP translation. It is also noteworthy that hnRNPF and hnRNPH, two ribonucleoproteins previously found to regulate the differential splicing of BCL-X into the anti-apoptotic protein BCL-XL or the pro-apoptotic proteins Bcl-XS (81) were found to associate with S6K2 in a mitogen-inducible manner (72). Hence, in addition to being able to regulate the translation of this protein, S6K2 may also be able to promote the preferential splicing of BCL-X toward BcL-XL.

Further work in U2OS osteosarcoma cells demonstrated that the Janus kinase TYK2 participated to the initially identified S6K2/BRAF/PKCε complex downstream of FGF2 signaling (82). In this cell system, TYK2 was required for the induction of the anti-apoptotic proteins BCL2 and MCL1 and for the promotion of cell survival in response to this growth factor. Whether this finding extends to lung, or other, cancer cells in which FGF2 signaling is relevant to the development of chemoresistance is at this point unknown. However, it suggests that inhibition of TYK2 may represent a new therapeutic strategy to target drug resistance downstream of FGF2 signaling.

The role of S6K2 in the control of cell survival may extend to neurodegenerative disorders. Frontotemporal dementia (FTD) is a form of pre-senile dementia associated with focal atrophy of the frontal or temporal lobes accompanied by deficits in cognition, behavior, and language. Mutations in progranulin (PGRN), a protein involved in cell growth and survival (83–85), are a common cause of FTD (86). Human neurons obtained from FTD patients with mutant PGRN were shown to have reduced cell viability that correlated with a downregulation of S6K2 transcription (87). All these changes were rescued by expression of wild-type PGRN, directly linking this factor with expression of S6K2. This link may have far wider relevance as serum levels of PGRN are a clinically significant predictive marker for recurrence in patients with HR-positive breast cancer during adjuvant tamoxifen therapy (88). Also, high PGRN expression levels correlate with an aggressive phenotype in cancer cell lines and clinical specimens from gliomas, breast, ovarian, and renal cancers (83). However, the link between PGRN and S6K2 in these settings is yet to be established.

### S6K2 and cognition

Behavioral analysis of S6K knockout mice highlighted non-overlapping cognitive functions for S6K1 and 2. S6K1 and S6K2-deficient mice were tested for contextual fear memory, conditioned taste aversion, Morris water maze acquisition, exploratory behavior, and long-term potentiation (89). Deficit in individual S6K isoforms resulted in distinct patterns of behavioral modifications with S6K1 being associated with the most pronounced phenotype. While both isoforms participated to contextual fear memory, conditioned taste aversion, and early-phase long-term potentiation, S6K2 deficit impacted particularly on long-term contextual fear memory and reduced latent inhibition of conditioned taste aversion. However, S6K2^−/−^ mice displayed normal spatial learning in the Morris water maze.

## S6K2 as a Therapeutic Target for Cancer

### Important considerations in the designs of S6K2 targeting therapy

Since knockout mice for S6K2 have shown that this kinase was dispensable for normal development and homeostasis, this kinase may represent an excellent therapeutic target for cancer. However, several lines of evidence suggest that S6K2 targeting should be selective and not impinge on S6K1 activity. Indeed, as indicated above, unlike S6K2 single-knockout animals, S6K1^−/−^/S6K2^−/−^ mice displayed perinatal lethality (47), suggesting that acute inhibition of both isoforms simultaneously in the absence of compensatory mechanisms may be deleterious to normal homeostasis. This is confirmed by work done in *Drosophila* where disruption of the unique S6K gene, *dS6K*, results in the death of the majority of flies at the larval stage or early pupation (90). Moreover, the S6Ks have been involved in negative feedback loops that regulate the PI3K and mTOR pathways. First, it was found that S6K1 and S6K2 phosphorylate IRS-1 on serine 302, a site adjacent to its PTB domain. This phosphorylation event inhibits the binding of IRS-1 to the insulin receptor, preventing further stimulation of the PI3K pathway by insulin (91, 92). Conversely, silencing of S6K1 using siRNAs was shown to decrease IRS-1 phosphorylation on several serine residues leading to an increase in PI3K/AKT signaling (93). In addition to targeting IRS-1, it has recently been revealed that S6K1, but not S6K2, modulates the activation of AKT by phosphorylating the mTORC2 complex component RICTOR on T1135 (49, 94, 95). This phosphorylation event does not seem to directly modulate the kinase activity of the mTORC2 complex. However, expression of a phospho-deficient mutant version of RICTOR promoted phosphorylation of AKT on S473, a site associated with activation of this kinase (49). Hence, considering the well-documented function of AKT in tumorigenesis (96) and the lethality of S6K double-knockout animals, a therapeutic strategy targeting both S6K isoforms in cancer patient may not be advisable. This hypothesis is further supported by results obtained using the pan-S6K inhibitor, LYS6K2, which increases basal and mitogen-induced AKT phosphorylation in treated cell lines (97). Conversely, the use of rapamycin analogs to inhibit the mTOR pathway, an approach that has been the subject of numerous cancer clinical trials (98), would not be appropriate in targeting the biological effects of S6K2 as some of the functions of this kinase are resistant to this inhibitor (13, 42, 79). Hence, it would be highly desirable to develop S6K2-specific compounds. However, at present, no drug discovery project has attempted to specifically target S6K2.

### Small-molecule ATP competitors

While S6K kinase inhibitors do exist, these are either pan-S6K compounds that also target other AGC kinases [e.g., Ref. (99, 100)] or compounds that show relative selectivity for S6K1 (101, 102). It was initially thought that the high degree of identity between the kinase domains of S6K1 and S6K2 would prevent the development of S6K2-selective kinase inhibitors. However, the existence of “selective” S6K1 inhibitors (101, 102) together with data acquired through 3-D modeling suggest that the development of such compounds would be possible. Indeed, comparison of the crystal structure of the kinase domain of S6K1 bound to Staurosporine (103) with the 3-D model of the same region in S6K2 reveals that the two kinases may differ in their contact with this inhibitor. This divergence occurs at the level of cysteine 150, a residue within the hinge region of S6K2 and may be exploitable to tweak selectivity of pan-S6K compounds toward this isoform. In support for this idea, the modulation of interaction of chemical compounds with the hinge region has previously been exploited to introduce selectivity among kinase inhibitors (104). Nevertheless, the high level of homology between the kinase domains of AGC kinases family members will always render the broader selectivity of these compounds hard to secure, especially *in vivo* where their intracellular concentration cannot be easily controlled. Hence, other approaches for the selective inhibition of S6K2 should also be considered.

### Protein–protein interaction inhibitors

We propose that the development of protein–protein interaction inhibitors would be a more appropriate strategy to specifically target S6K2. Indeed, drug discovery efforts into protein–protein interaction inhibitors have multiplied in the last few years [e.g., Ref. (105–109) to only cite a few] and some have already yielded compounds that entered the clinic (105). The heightened interest in this approach has been fueled by the relative lack of success of small-molecule kinase inhibitors in the clinic, together with the development of novel bioinformatics tools to predict disruption of protein–protein interactions (110, 111). We and others have performed co-purification of S6K2 with its interacting partners downstream of mitogen stimulation in various cell lines [(69, 72) and unpublished], an effort that resulted in the identification of S6K2-specific interactors that regulate the biological functions of this kinase (42, 69, 72). For instance, interaction of S6K2 with BRAF and PKCε was shown to regulate the anti-apoptotic function of this kinase while that of S6K2 and hnRNPF modulated its effect on the cell cycle. Hence, one could envision drug discovery efforts aimed at targeting these identified interactions.

### Allosteric S6K2 inhibitors

Two more characteristics of S6K2 may be potentially exploitable for the development of selective compounds. The first is its C-terminal domain which differs significantly from the same region in S6K1 and is predicted through 3-D modeling to be fairly unstructured and exposed. This region could be used to fish out interacting compounds that may, through binding, alter the 3-D conformation of S6K2. As this region also contains the NLS for S6K2, interacting compounds may also interfere with its sub-cellular localization. The second S6K2 characteristic is its higher reliance for activation on the MEK/ERK-mediated derepression from the pseudo-substrate domain. Compounds that would bind to the kinase extension domain or hinder access to the proline-directed phosphorylation sites may prevent S6K2 from exposing the serine/threonine sites responsible for its activation to upstream kinases.

## Conclusion

It has now been 15 years since the cloning of S6K2. However, many aspects of the regulation and biological functions of this enzyme are still a mystery. Indeed, the high degree of homology between S6K1 and S6K2 led us to bias most of our research toward the first of these two isoforms, as these were thought to have identical roles. Instead, it is now becoming clear that these enzymes have distinct biological functions mediated by their distinct repertoires of substrates and interactors. Our limited knowledge of S6K2 suggests that this isoform may play a particularly important role in the pathobiology of cancer and targeting this isoform could provide a therapeutic benefit in patients. However, this will most probably require the development of S6K2 isoform-selective compounds that exploit its known specific protein–protein interactions and downstream substrates that mediate its functions. The time has come to embrace S6K2, recognize the biological diversity that this enzyme introduces into the S6 kinase pathway and exploit this information for new therapies.

## Conflict of Interest Statement

The authors declare that the research was conducted in the absence of any commercial or financial relationships that could be construed as a potential conflict of interest.
